# Machine Learning Identification of Pro-arrhythmic Structures in Cardiac Fibrosis

**DOI:** 10.3389/fphys.2021.709485

**Published:** 2021-08-13

**Authors:** Radek Halfar, Brodie A. J. Lawson, Rodrigo Weber dos Santos, Kevin Burrage

**Affiliations:** ^1^IT4Innovations, VSB-Technical University of Ostrava, Ostrava, Czechia; ^2^Centre for Data Science, School of Mathematical Sciences, Queensland University of Technology, Brisbane, QLD, Australia; ^3^ARC Centre of Excellence for Mathematical and Statistical Frontiers, School of Mathematical Sciences, Queensland University of Technology, Brisbane, QLD, Australia; ^4^Graduate Program in Computational Modeling, Universidade Federal de Juiz de Fora, Juiz de Fora, Brazil; ^5^Department of Computer Science, University of Oxford, Oxford, United Kingdom

**Keywords:** machine learning, neural networks, fibrosis, cardiac electrophysiology, arrhythmia, monodomain model, re-entry, unidirectional block

## Abstract

Cardiac fibrosis and other scarring of the heart, arising from conditions ranging from myocardial infarction to ageing, promotes dangerous arrhythmias by blocking the healthy propagation of cardiac excitation. Owing to the complexity of the dynamics of electrical signalling in the heart, however, the connection between different arrangements of blockage and various arrhythmic consequences remains poorly understood. Where a mechanism defies traditional understanding, machine learning can be invaluable for enabling accurate prediction of quantities of interest (measures of arrhythmic risk) in terms of predictor variables (such as the arrangement or pattern of obstructive scarring). In this study, we simulate the propagation of the action potential (AP) in tissue affected by fibrotic changes and hence detect sites that initiate re-entrant activation patterns. By separately considering multiple different stimulus regimes, we directly observe and quantify the sensitivity of re-entry formation to activation sequence in the fibrotic region. Then, by extracting the fibrotic structures around locations that both do and do not initiate re-entries, we use neural networks to determine to what extent re-entry initiation is predictable, and over what spatial scale conduction heterogeneities appear to act to produce this effect. We find that structural information within about 0.5 mm of a given point is sufficient to predict structures that initiate re-entry with more than 90% accuracy.

## 1. Introduction

According to the WHO, in 2016, 17.9 million people worldwide died of cardiovascular diseases (31% of all deaths). These diseases are the most common cause of death in the world. Although the function and dysfunction of the heart have been extensively studied, the sheer complexity of the spatiotemporal dynamics underlying its electrical signalling process leaves much still poorly understood. This is particularly true when complicating factors are present, such as cardiac fibrosis.

Cardiac fibrosis, the over-activity of fibroblasts in the heart, poses significant health risks (Hinderer and Schenke-Layland, 2019). Fibroblasts deposit extracellular matrix proteins that can separate myocytes, resulting in tortuous paths of activation that increase the risk of signalling malfunctions. This risk depends critically on the extent and arrangement of afflicted tissue, but this dependency is intricate and very difficult to quantify. Efforts have been made to classify different types of fibrotic patterning with the suggestion that might help stratify risk (de Jong et al., 2011) but with little attempt to explain why or how these different types of pattern present different levels of risk. A separate approach focuses on small-scale structures that produce key behaviours underlying re-entry and arrhythmia. The pro-arrhythmic mechanisms of fibrosis are well understood (Nguyen et al., 2014), but the precise patterns that do or do not trigger those mechanisms are not well understood. The computational simulation presents a powerful tool for investigating these structures mechanistically, and machine learning (ML) provides the opportunity to automate identification.

In this study, we consider the risk of re-entry posed by many different fundamental structures of fibrosis. The specific pattern of fibrosis plays two important roles in the promotion of re-entry or micro-re-entry: through re-entrant paths within the damaged region that are long enough to accommodate the wavelength of the propagating action potential (AP) and by the presence of structures that facilitate one-way block of AP propagation. We concentrate on the latter, that is, structures that selectively block conduction, for example, permitting conduction in one direction but not the other. This phenomenon of a *unidirectional block* is a critical precursor to re-entry (Quan and Rudy, 1990).

Computational studies have successfully reproduced re-entries from fibrosis for different types of diseases, such as atrial fibrillation (Alonso et al., 2016; Vigmond et al., 2016), myocardial infarction (Sachetto Oliveira et al., 2018a), and many other pathologies related, for instance, to hypoxia and fibrosis including hypertrophic cardiomyopathy, hypertensive heart disease, recurrent myocardial infarction, obstructive pulmonary disease, obstructive sleep apnoea, and cystic fibrosis (Sachetto Oliveira et al., 2018b). However, as we do not know which kind of patterns within the fibrotic substrate are pro-arrhythmic, these studies depend on the generation of hundreds of thousands of fibrosis patterns, followed by Monte Carlo simulations and statistical analysis. These studies have investigated, for example, the probability of re-entry as a function of the fraction of damaged tissue. Nevertheless, the kind of patterns that facilitate unidirectional blocks and how often these patterns are present in damaged tissues are important open questions.

Machine learning (ML), as with most fields, has begun to see a considerable application to cardiac electrophysiology. These include automated extraction of subtle information from the electrogram (Yang et al., 2018; Mincholé et al., 2019) and the identification of promising targets or success rates for ablation (Zahid et al., 2016; Muffoletto et al., 2019, 2021; Shade et al., 2020). In this study, we generate a large number of different realisations of fibrotic arrangement corresponding to significantly damaged tissue and then apply a single stimulus originating from many different points. This creates a rich dataset of structures that give rise to re-entry. We then isolate regions of selective block and train a classifier model that identifies with high accuracy whether a given pattern of fibrosis generates this pro-arrhythmic behaviour. Importantly, this successful classification is a first step to address fundamental questions relating anatomical heterogeneity to re-entry risk, and over what spatial scale these effects manifest.

## 2. Materials and Methods

### 2.1. Simulation of Cardiac Activity

We simulate cardiac activity inside the regions afflicted with fibrosis, examining the patternings of obstacles to conduction that initiate re-entries sustained inside these fibrotic regions. These micro-re-entries cause fibrotic regions to act potentially as ectopic pacemakers that drive tachycardia or other arrhythmia (Hansen et al., 2015). As our focus is on the initiation and immediate sustainment of re-entry, we do not simulate how waves of activation produced by a fibrotic region interact with healthy surrounding tissue, nor do we consider scenarios such as fast pacing that indicate the existence of prior signalling dysfunction.

**Figure 1:**
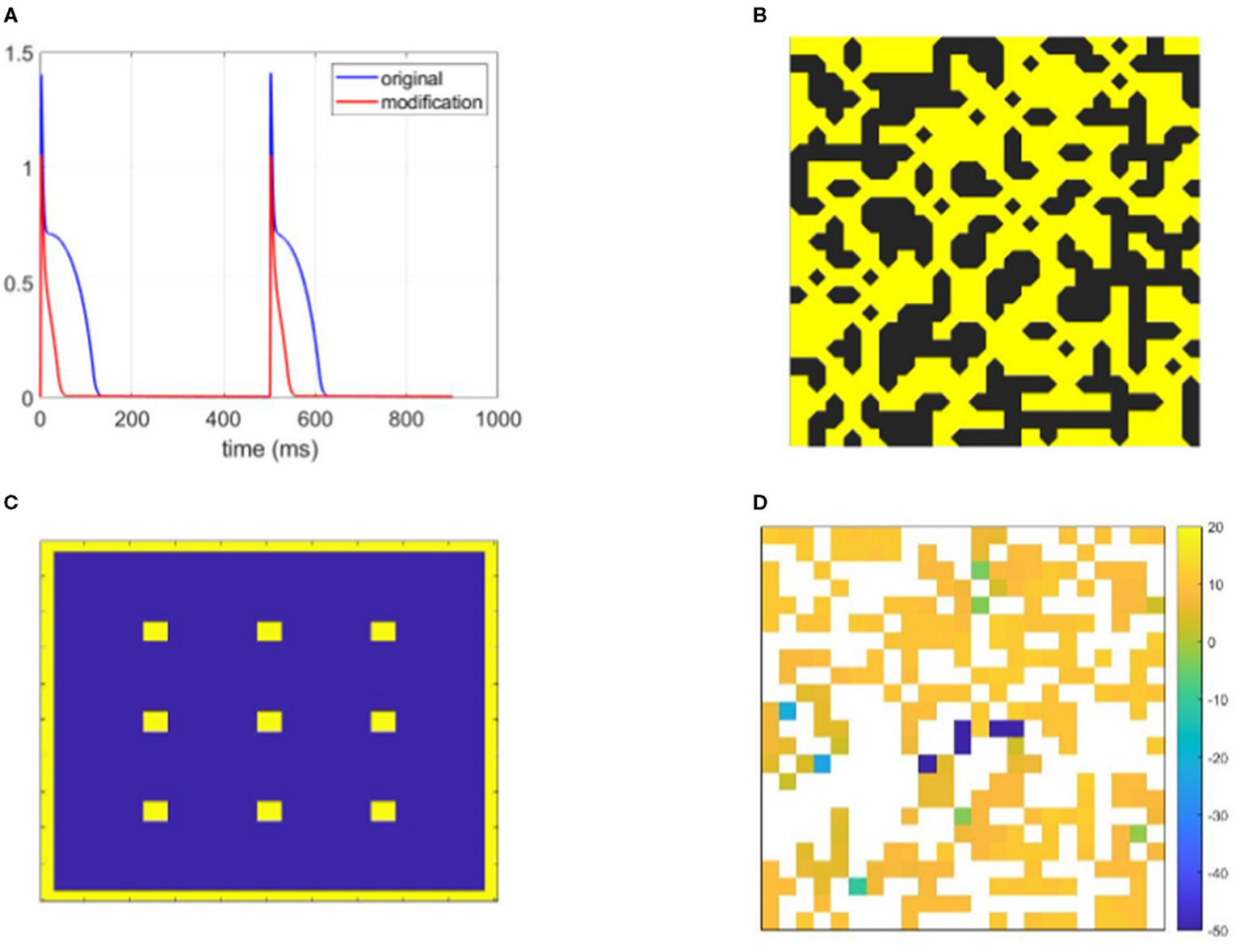
Graphical demonstration of some of the methods used in this study. **(A)** The action potential (AP) of the Bueno-Orovio-Cherry-Fenton (BOCF) model modified to represent strongly fibrosis-afflicted tissue (parameters in Table 1), and the original BOCF model. Remodelled myocytes repolarise very rapidly with a triangular-shaped AP. **(B)** An example fibrotic structure, visualised to highlight the ‘diagonal' connectivity inherent to placing nodes on element vertices. **(C)** The stimulus locations (yellow) used across separate simulations to generate wavefronts travelling in different directions and hence bolster identification of structures that produce re-entry. **(D)** Re-entry vulnerability index (RVI) values observed for the structure pictured in **(B)**, showing the identification (by significantly negative value) of locations that demonstrate selective conduction block.

Cardiac electrophysiological dynamics were simulated using the monodomain formulation (Sundnes et al., 2006),

(1) $$\begin{array}{r} C_{m}\frac{\partial V}{\partial t}=\nabla \cdot \left(D\nabla V\right)-I_{\text{ion}}, \end{array}$$

which treats cardiac cells as capacitive and hence describes the change in their membrane potential in terms of the current that flows diffusively to/from neighbouring cells through gap junctions and by ion transport through the ion channels of the cell membrane. We use a capacitance density of *C*_*m*_= 1 μF m-2 and electrical conductivity *D* = 2.5 × 10^−4^ mS. Cell APs were simulated using the Bueno-Orovio–Cherry–Fenton (BOCF) model, a reduced model that nevertheless accurately captures the most important electrophysiological dynamics of ventricular myocytes (Bueno-Orovio et al., 2008). To represent the effects of significant tissue damage on APs Shaw and Rudy (1997); Sachetto Oliveira et al. (2018b), we modified model parameters to shorten AP duration (APD) to approximately 50 ms (see Figure 1A and Table 1). This results in a conduction velocity of 23 cm s-1, reflecting the decreased gap junction functionality in diseased tissue (Duffy, 2012; Nguyen et al., 2014).

**Table 1:** The parameters of the Bueno-Orovio-Cherry-Fenton (BOCF) model, modified to represent cardiac tissue with significant fibrosis.

**Parameter**	**Value**	**Parameter**	**Value**	**Parameter**	**Value**
*C*_*m*_	1	$\tau_{v}^{+}$	1.4506	τ_*s*1_	2.7342
*u*_*v*_	0.3	$\tau_{v1}^{-}$	60	τ_*s*2_	16
$u_{v}^{-}$	0.006	$\tau_{v2}^{-}$	1150	τ_*fi*_	0.11*C*_*m*_
*u*_*w*_	0.13	$\tau_{w}^{+}$	200	τ_*si*_	2.8
$u_{w}^{-}$	0.03	$\tau_{w1}^{-}$	60	τ_*so*1_	30.0181
*u*_*o*_	0.006	$\tau_{w2}^{-}$	15	τ_*so*2_	0.9957
*u*_*s*_	0.9087	τ_*w*_∞__	0.07	*k*_*s*_	2.0994
*u*_*so*_	0.4	τ_*o*1_	400	$k_{w}^{-}$	65
*u*_*u*_	1.2	τ_*o*2_	6	*k*_*so*_	2.0458
$w_{\infty }^{*}$	0.94				

*Parameter notation is that of Bueno-Orovio et al. (2008)*.

Simulations were carried out in two-dimensional, 2 × 2 cm slices of isotropically conductive cardiac tissue. We chose a larger amount of tissue than the minimum needed to support re-entry as reported for these types of conditions (0.7 × 0.7 cm; Sachetto Oliveira et al., 2018b), so as to increase the number of re-entries present in our generated data. The effect of fibrosis on conduction was represented by the presence of non-conducting obstacles (for example collagen), a common approach taken for both ventricular tissue (Ten Tusscher and Panfilov, 2007; McDowell et al., 2011) and atrial tissue (Cherry et al., 2007; McDowell et al., 2015), as well as highly-detailed microscopic models of cardiac tissue where cells are disconnected by barriers or dead cells (Jacquemet and Henriquez, 2009; Hubbard and Henriquez, 2014; Gouvêa de Barros et al., 2015). This approach is in contrast to approaches that represent fibrotic obstacles indirectly through modifications to conductivity in afflicted areas, often in response to imaging data informing fibroblast density (Zahid et al., 2016; Roy et al., 2020).

Obstacles were seeded randomly through the domain by randomly replacing each grid element with a non-conductive element with some fixed probability ρ, a typical approach used for modelling diffuse fibrosis (Kazbanov et al., 2016). We did not explicitly consider the other types of fibrotic microtexture (such as compact or patchy fibrosis de Jong et al., 2011). However, by choosing ρ~0.5 and simulating many different realisations, we have considered a very broad range of patterns on the fine-scale that we analyse in this study. It is worth noting that other types of fibrotic patterning could be directly incorporated into our machine learning workflow through recent techniques for computer generation of large numbers of realisations of different fibrotic patterns (Clayton, 2018; Jakes et al., 2019).

Equation (1) was discretised using a vertex-centred control volume finite element method that integrates bilinear interpolants over the square-shaped elements. This generates a non-diagonal mass matrix and significantly reduces discretisation error in this sharp-fronted wavefront setting (Pathmanathan et al., 2012). For a vertex-centred mesh where nodes are at element vertices, excitation can still propagate through the “crack” between diagonally opposed obstructions, owing to a node being there. As such, to make our visualisations of fibrotic structures more intuitive, we display fibrotic obstructions such that these diagonal connections are respected (Figure 1B). Timestepping used the second-order generalisation of the Rush–Larsen method put forward by Perego and Veneziani (2009), with Δ*t*= 0.05 ms. Simulations continued until all cardiac activity died out, or *t*= 2 s was reached. These simulations were carried out on the Barbora supercomputer (Czech Republic).

### 2.2. Re-entries and Conduction Block

**Figure 2:**
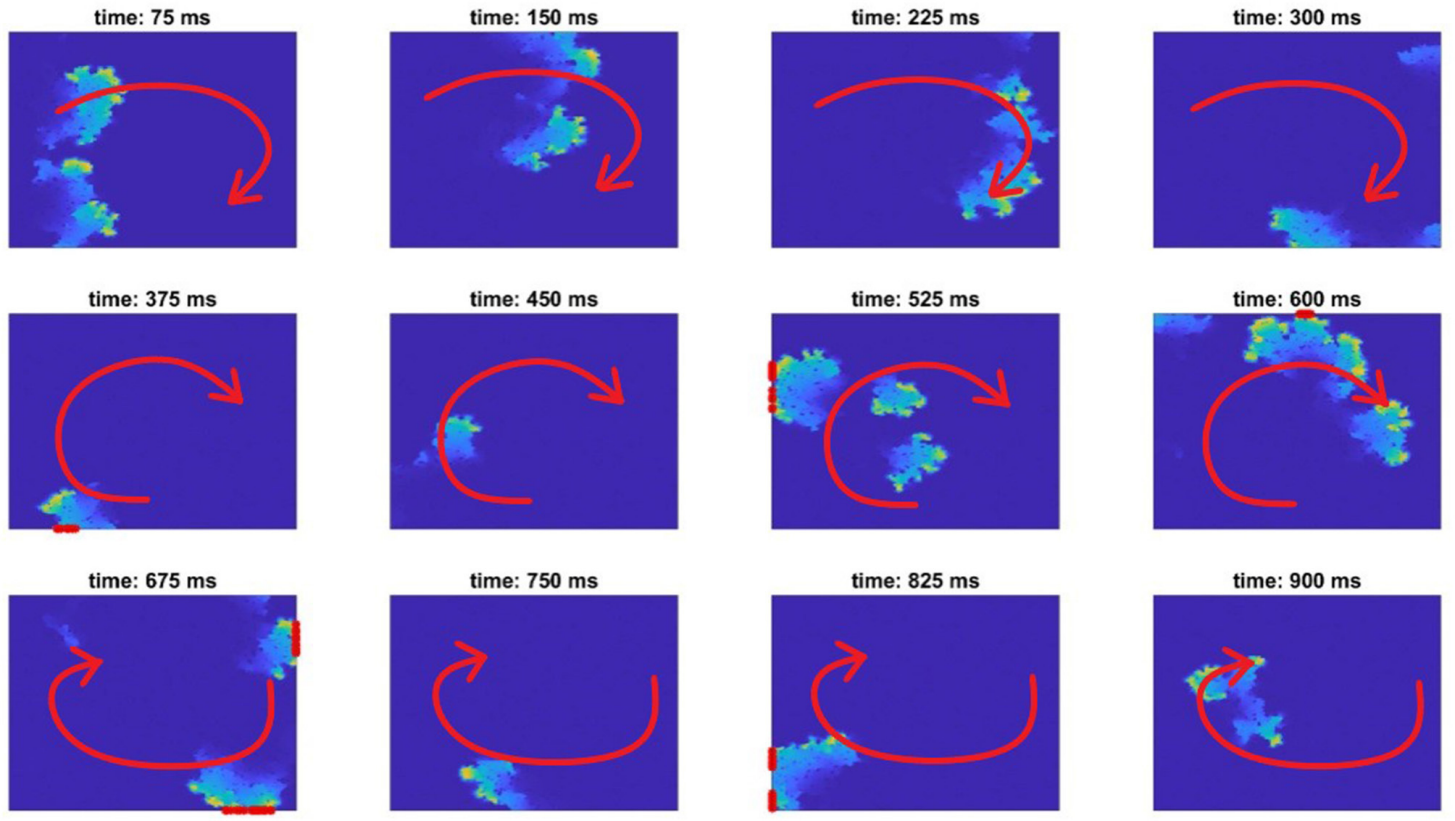
A re-entry formed in fibrotic tissue (red arrow indicates the direction of AP propagation), and its detection. An AP initialised on the left border propagates through the tissue, failing to conduct through the bottom passage. Then, when the excitation turns around (about 250 ms), it transmits through this bottom passage and successfully re-emerges into the remainder of tissue, forming a re-entry (about 375 ms). Only re-entries that might escape back into the tissue surrounding the afflicted region are counted, as detected by nodes sitting on the boundary of the domain being activated more than one time (marked with a red asterisk on the boundaries).

Our study concentrates solely on the effect of structure on the initiation of re-entrant patterns of activation. As such, each individual simulation used only one stimulus pulse so as to preclude other conflating factors such as repolarisation heterogeneity in scarred tissue (Gough et al., 1985). However, to maximise the opportunity to identify pro-arrhythmic structures, we increased robustness to specific propagation directions and patterns of activation by separately using 13 different stimulus sites for each fibrotic realisation (Figure 1C). To obtain sufficient data featuring re-entry, a sweep through values 0.4 ≤ ρ ≤ 0.6 was first used to determine those extents of fibrosis prone to re-entry. For each density value considered, 50 different realisations of fibrosis were created. Re-entry was detected by the activation of any boundary nodes more than one time (Figure 2), capturing ectopic waves that successfully escape the fibrotic region being simulated. A realisation of fibrotic structure that generated a re-entry for any of the possible stimulus sites was then labelled as a substrate for re-entry.

Following initial observations, our high-throughput simulation protocol concentrated on the range ρ ∈ [0.46, 0.50] as the values most prone to re-entry. For each ρ value in this range (in increments of 0.01), an additional 800 fibrotic patterns were created, and the same simulation protocol as above then applied to each. Table 2 summarises the size, and basic qualities, of the resulting data.

**Table 2:** Summary of the simulations performed, and the resulting data used for machine learning (ML) (using one structure size as an example).

65,650	Total simulations
3,902	Simulations featuring a re-entry (that reached the boundary)
5,050	Unique arrangements of fibrosis
1,907	Fibrotic arrangements that generated re-entry
228,659	11 × 11 binary patterns exhibiting selective block
228,571	11 × 11 binary patterns not exhibiting selective block

**Figure 3:**
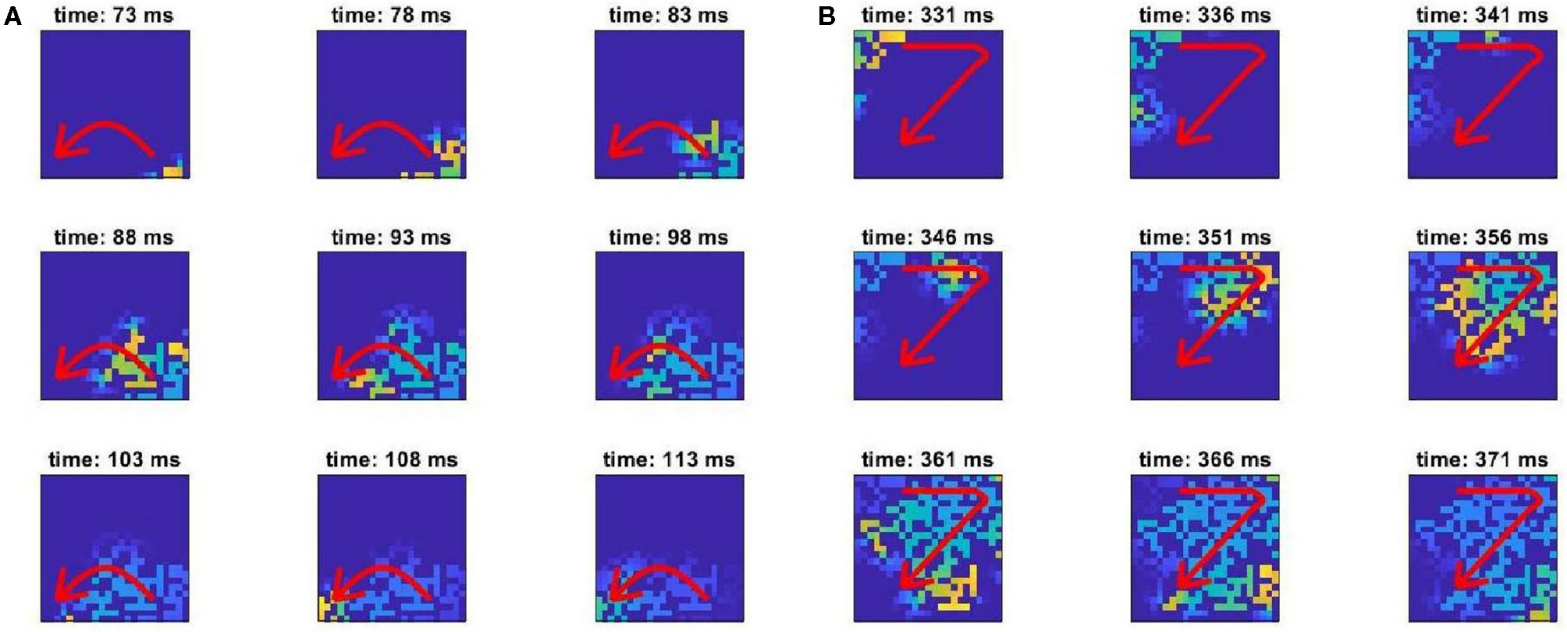
Snapshots of AP propagation demonstrating an event of the unidirectional block. Visualised is one section of the full fibrotic region, detected by our RVI-based approach. The brightness of colour indicates level of activation, and the red arrows indicate the overall direction of propagation. **(A)** The wave propagates from the bottom-right to the bottom-left corner of the section, attempting also to propagate through the central passage but failing due to an imbalance between excited and excitable tissue. **(B)** When the wavefront later propagates through the top portion of this structure, it is able to successfully propagate downwards through the central passage, re-entering into the tissue in the bottom portion.

To detect specific micro-structures that promote re-entry, we used the re-entry vulnerability index (RVI) (Orini et al., 2017; Orini et al., 2019). This index calculates the difference in activation time for a node and the repolarisation time of its neighbours, and hence indicates potential for re-entry formation (Figure 1D). In particular negative values occur when a neighbouring node has already activated and repolarised when a node first activates, allowing the node to spread its activation back to that neighbour and potentially much more of the tissue. This scenario arises when conduction blocks despite the existence of waiting excitable tissue, for example, due to excessive electrotonic loss (Nguyen et al., 2014). An example of conduction dying out due to source-sink mismatch, only for wave propagation to succeed in travelling through the same structure from a different direction, is provided in Figure 3.

Significantly negative RVI values further indicate a likelihood that surrounding tissue will also be ready to excite, increasing the risk that a re-entrant event develops into an ectopic wavefront significant enough to escape and hence trigger extrasystole. We, therefore, find all locations that exhibited RVI values below a threshold RVI ≤ −50. When multiple locations were detected together as a contiguous group, these were simplified to a single location. Around each detected site, the patterning of fibrosis (as an array of binary values) was extracted, and labelled as a “discriminative” structure, reflecting its inconsistent passing along the excitation dependent on wavefront direction or other conditions. To complete the dataset, this set of structures was complemented by a set of ‘indiscriminate' structures of the same size, selected by finding locations that satisfied two conditions. First, indiscriminate structures have to be activated (at least 40% of their constituent excitable tissue), so that their effects on wavefront propagation had been tested by the simulation they came from. Second, indiscriminate structures could not contain any locations identified by RVI values under the threshold as discriminative.

### 2.3. Pattern Classification

To explore how much information regarding re-entry risk is contained in the patterning of fibrosis, we considered the ability of neural networks (NN) to successfully classify different structures as discriminative about excitation transfer or not. The datasets were made balanced by detecting and adding indiscriminate structures until these were the same in number as the discriminative structures. As each structure is a binary mask, they can simply be converted to a vector of 0 and 1 values to serve as input to an NN. The NN then outputs a single value indicating a category to which structure belongs (discriminative or not).

A variety of NN architectures were considered, using densely interconnected layers and zero to four hidden layers. Layer size varied from 100 to 1,200 neurons. All NN training and evaluation used the Keras application programming interface (API) (Chollet, Francois et al., 2015), a popular Python library for machine learning. We used the *Adam* optimiser with a binary cross-entropy loss function to optimise the neural network. The rectified linear activation function (*ReLU*) activation function was used in the inner layers and a sigmoid activation function in the outer layer. To explore the spatial scale on which patterning acts to create selective block of conduction and hence re-entry, we also considered the ability to identify selectively blocking patterns when working with structures of various sizes. In particular we take the element identified *via* RVI as the centre of a square binary pattern, with side lengths varying from 5 elements (0.5 mm) to 23 elements (2.3 mm).

## 3. Results

### 3.1. Preliminary Results

**Figure 4:**
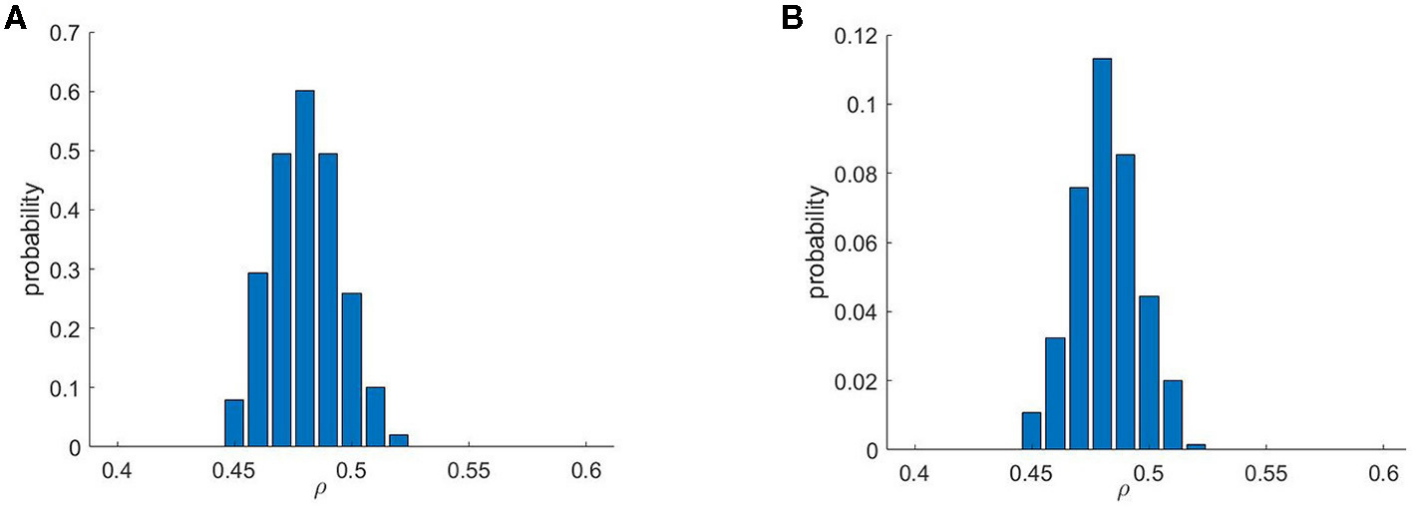
Re-entry formation depends critically on the amount of fibrotic obstructions. Only a specific range of values of ρ, the probability that any individual mesh element is obstructed, permits re-entry formation. Shown are the probabilities that a given fibrotic realisation produced a re-entry for **(A)** at least one stimulus scenario and **(B)** for an individual stimulus scenario. A comparison of these two histograms highlights the importance of considering multiple stimulus locations when evaluating a structure for potential as an arrhythmic substrate.

As briefly mentioned in Methods, re-entries were found to appear only within a rather selective range of ρ values (Figure 4), matching observations of previous studies considering micro re-entry in untextured fibrosis (Sachetto Oliveira et al., 2018a,b). This effect is caused by the requirement for both a sufficient amount of obstruction to create the structures that produce a source-sink mismatch, and a sufficiently conductive structure for any resulting re-entrant event to successfully reach the domain boundary and hence produce an ectopic beat. This balance is strongly related to the percolation threshold, and we note that the critical range of 0.45 ≤ ρ ≤ 0.52 for re-entry is here larger than in the previous studies, as vertex-centred meshes are naturally more conductive. Figure 4 also compares the chance of re-entry for any individual simulation (one stimulus site), with the chance per pattern realisation (for at least one re-entry across all stimulus sites). Even given that a structure can produce re-entries that escape the fibrotic region, only very few choices of stimulus location result in this behaviour, demonstrating a significant sensitivity to activation pattern.

**Figure 5:**
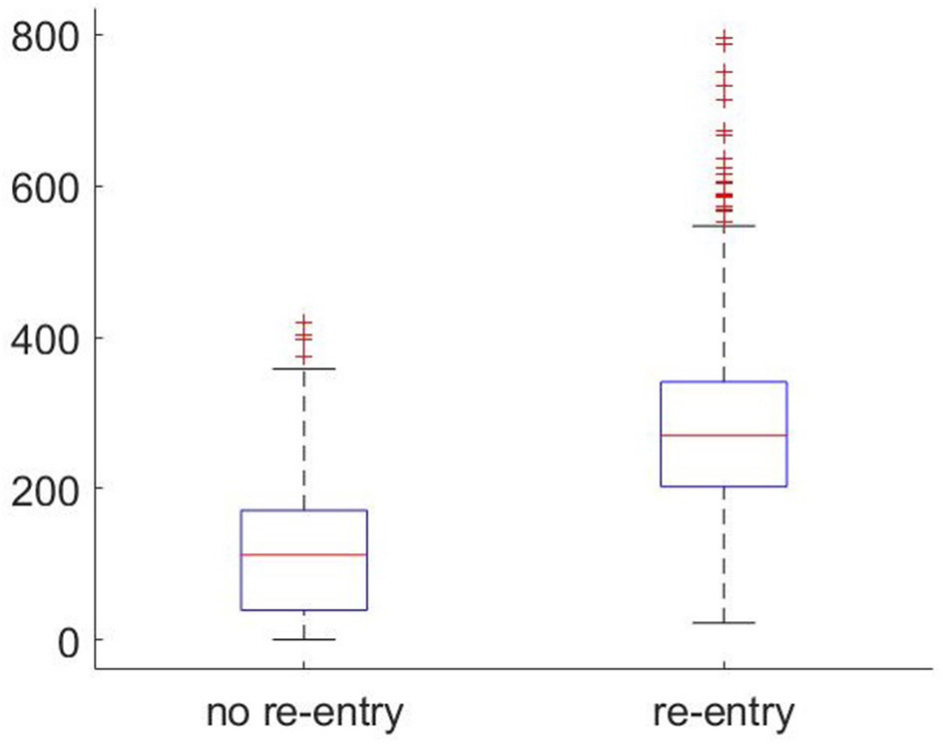
Boxplots showing the frequency of microstructures that selectively block condution (as detected by significant negative RVI) occurring in large-scale fibrotic realisations that did or did not exhibit re-entry. The higher the number of such discriminative structures found, the more likely a re-entrant AP will survive and then escape into the surrounding tissue.

Figure 5 compares the frequency with which selectively blocking micropatterns were identified across the large-scale fibrotic realisations (4 cm2) that did or did not result in re-entry. The cases exhibiting re-entry showed on average more than two times as many selectively blocking sites than those that did not. This confirms the intuition that the presence of microstructures that may initiate re-entry correlates significantly with the overall risk posed by a fibrotic region. However, even those realisations that did not produce re-entry under any stimulus scenario still produced many individual events of unidirectional or other selective block of conduction. This shows that the mutual spatial arrangement of these initiator patterns, and the larger-scale structure more generally, is also critical to the formation of re-entries that persist and escape into the surrounding tissue. Notably, there exists a positive feedback effect when it comes to simply counting detected discriminative microstructures, and as once a re-entry has successfully formed, there is an additional opportunity for repolarisation heterogeneity to produce further block events in vulnerable microstructures.

**Figure 6:**
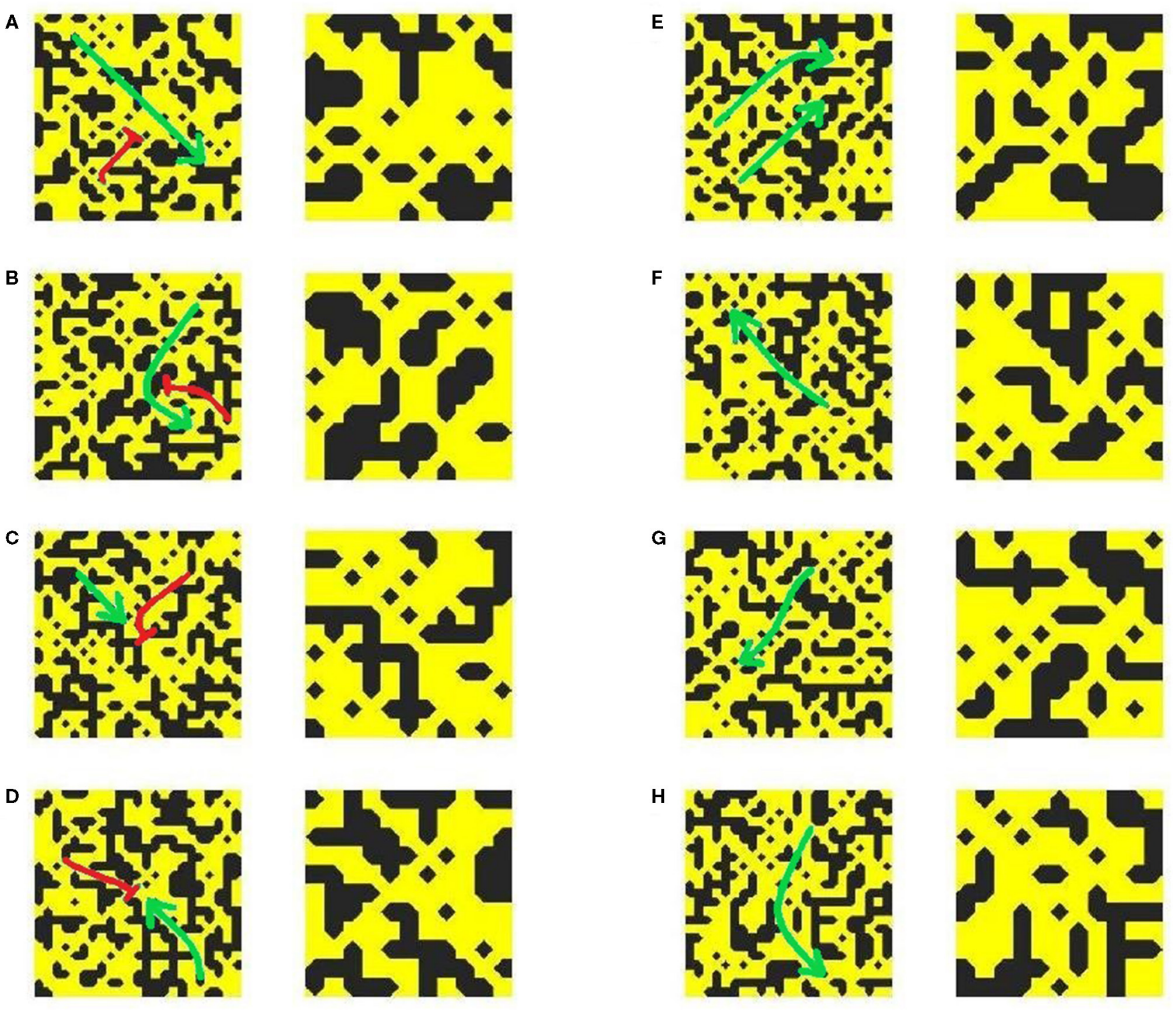
Examples of pro-arrhythmic **(A–D)** and non-arrhythmogenic **(E–H)** micropatterns (23 × 23 elements), and a close-up view of the structure at their centre. Green arrows indicate the directions of AP propagation, with red flat arrowheads indicating conduction block.

Individual examples of micropatterns capable or incapable of initiating re-entry, as detected by our methods, are presented in Figure 6. As shown by the arrows indicating the direction of AP propagation (or block), the pro-arrhythmic patterns (left side) all result in unidirectional block. Examining the fine-scale structures that produce this effect reveals broad correspondence to the AP emerging from thin passages into larger regions of open tissue. This is the classical example of structural heterogeneity producing unidirectional block through source-sink mismatch (Ciaccio et al., 2018). However, the rich diversity of patterning in these structures and the presence of visually similar arrangements in the structures observed to permit normal conduction (right side of figure) highlight the difficulty of differentiating by eye alone patterns that may or may not initiate re-entry. This motivates the use of machine learning as a more accurate, and automated, means of carrying out this classification.

### 3.2. Classification of Micropatterns That Can Initiate Re-entry

The micropatterns that do or do not exhibit selective (unidirectional, or inconsistent) conduction block were learned by training a NN classifier, as described in Methods. Depending on the NN architecture and micropattern size, the overall accuracy of the classifier (as evaluated using unseen test data) ranged from approximately 75 to 91%. Specificity and sensitivity ranged from 74 to 91%, and the area under the receiver operating characteristic curve (ROC) curve ranged from 0.82 to 0.95. The dependence of performance on network architecture, for a fixed micropattern size, is summarised in Table 3, where it can be seen that maximal classification accuracy of 91% was obtained by using two hidden layers of 1,000 neurons each. This architecture strikes the balance between including enough neurons to capture the high complexity of the classification problem, and the risks of training difficulties or overfitting posed by a network with too many neurons. The classification problems using other micropattern sizes showed very similar relationships between accuracy and network architecture. In Table 4 is shown the confusion matrix of the NN for micropatterns of size 23 × 23, and 9 × 9. These results confirm that NN performance is balanced, that is, the NN can detect pro-arrhythmic as well as non pro-arrhythmic structures with the same accuracy.

**Table 3:** The resulting accuracy/area under the curve (AUC) of the neural network (NN) for the size of the micropattern 9.

		**Hidden layers**				
		**0**	**1**	**2**	**3**	**4**
Neurons in layer	100	0.758/0.837	0.791/0.871	0.804/0.881	0.809/0.887	0.817/0.891
	200	0.778/0.855	0.844/0.91	0.864/0.925	0.865/0.925	0.866/0.925
	400	0.81/0.884	0.893/0.937	0.886/0.938	0.895/0.941	0.882/0.933
	600	0.833/0.898	0.899/0.943	0.901/0.945	0.901/0.946	0.9/0.946
	800	0.848/0.907	0.894/0.938	0.9/0.945	0.904/0.947	0.894/0.938
	1000	0.855/0.915	0.904/0.946	0.911/0.952	0.909/0.951	0.903/0.948
	1200	0.856/0.915	0.908/0.947	0.91/0.95	0.905/0.946	0.897/0.947

**Table 4:** (A) The confusion matrix of the NN for 23 × 23 micropatterns, with four hidden layers and 800 neurons in each layer.

		**True state**	
		**Pro-arrhythmic**	**Not pro-arrhythmic**
**(A)**			
Prediction	Pro-arrhythmic	17,179	4,611
	Not pro-arrhythmic	4,616	17,174
**(B)**			
Prediction	Pro-arrhythmic	20911	2,090
	Not pro-arrhythmic	2,094	20,915

*(B) The confusion matrix of the NN for 9 × 9 micropatterns, using three hidden layers and 1,000 neurons in each layer*.

The classifier models with appropriate architectures obtain very good accuracy, considering they are attempting to identify a complex phenomenon such as unidirectional or otherwise selective block only from binary micropattern data. On one hand, we have considered many different patterns of activation (by using different choices of stimulus site) to generate these data, and so structures identified as pro-arrhythmic might still exist safely in a scar region if they never experienced waves travelling in the necessary direction to trigger the initial re-entry. On the other hand, structures identified as non-arrhythmogenic will have been subjected to multiple different AP propagation scenarios. This suggests that microstructures identified as indiscriminate could potentially be considered safe independent of the factor of wavefront direction.

Classifier accuracy also allows us to consider the information necessary in order to identify pro-arrhythmic micropatterns of obstruction. In this study, we have varied the size of these micropatterns, and thus can gain some understanding regarding the spatial scale on which the dynamics of unidirectional or selective block truly acts. On one hand, if the structures considered are too small to correctly identify the relevant source-sink interactions, accuracy will suffer due to this lack of requisite information. On the other hand, when redundant information is included by using a too large micropattern size, this only increases the dimensionality of the learning problem without supplying anything useful, and accuracy suffers due to the negatively shifted the balance between dimension and amount of training data.

**Figure 7:**
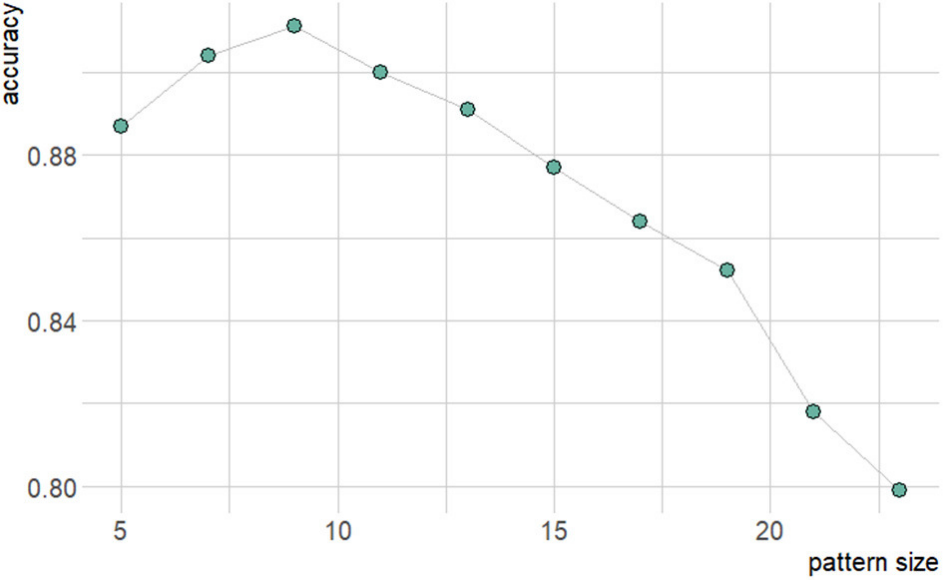
Graph of resulting accuracy dependence on micropattern size for two hidden layers and 1,000 neurons.

**Figure 8:**
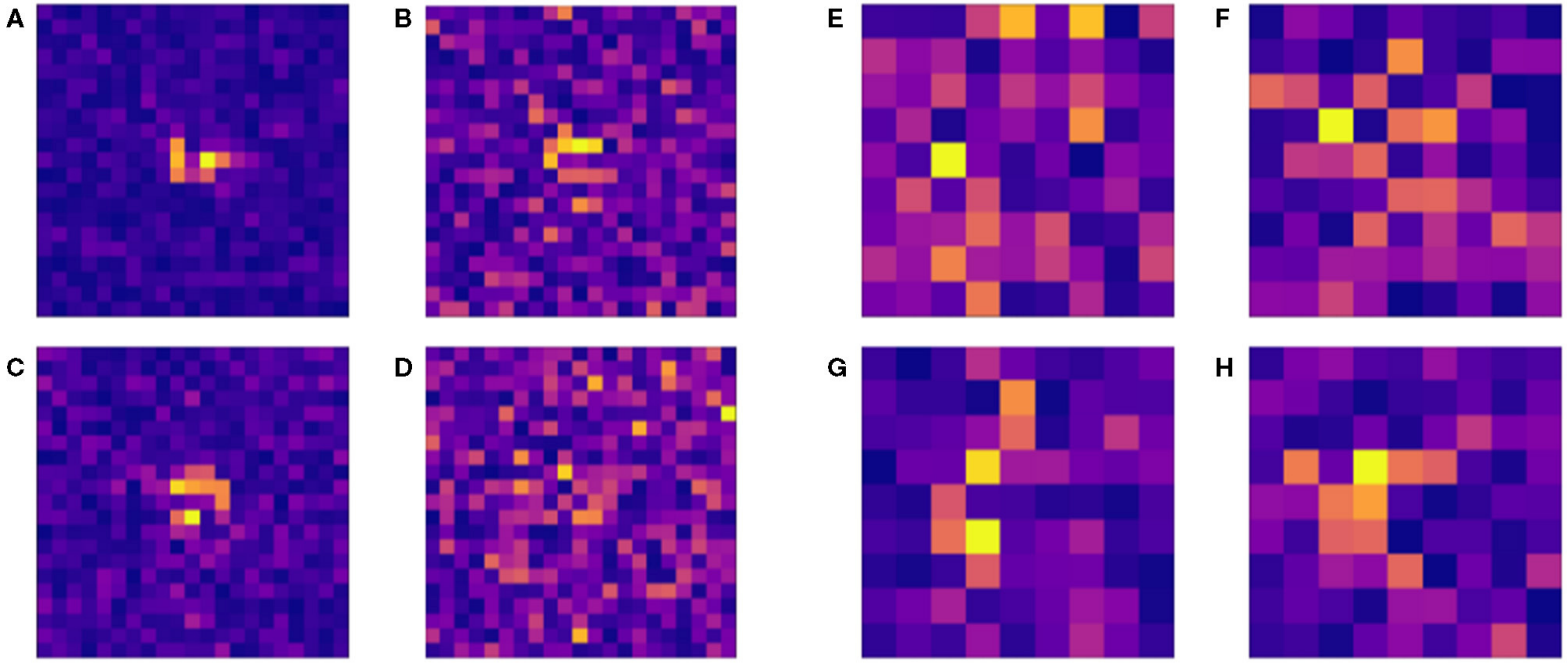
Example saliency maps for a selection of 21 × 21 **(A–D)** patterns classified by a neural network with zero hidden layers and 1,200 neurons in one layer and 9 × 9 patterns **(E–H)** with two hidden layers and 1,000 neurons in one layer. The lightness of grid sites indicates their level of contribution towards the decision of the classifier for the different micropatterns tested. In the case of the larger patterns **(A–D)**, site importance is concentrated around the centre of the pattern, whereas smaller patterns more consistently use sites throughout the pattern to evaluate a structure for selective conduction block. This supports the conclusion that the vast majority of these proarrhythmic phenomena take place on smaller spatial scales.

Figure 7 shows how changes to micropattern size impact the accuracy of the resulting classifier models. Accuracy peaks for patterns of size 9 × 9, suggesting that the balance of source-sink mismatch for a wavefront is meaningfully controlled by the surrounding structure on a length scale of about 0.4–1 mm. The larger end of this range arises from the observation that with increased amounts of training data, higher-dimensional datasets may have exhibited even higher classification accuracy. Saliency maps, which show the respective levels of contribution of the individual elements of a structure towards the resulting classification output by a NN, also showed a tendency to concentrate importance on a small central subsection of the larger micropatterns (Figure 8). This provides further evidence towards the conclusion that selective and unidrectional block events are governed by structure over only a small length scale.

### 3.3. Generalisation to New Data

In discussing classifier model accuracy, we have been referring to the performance of the model in classifying micropatterns not seen by it during the training process, but still sourcing from the same overall batch of simulations from which the training data were taken.

In this study, we test the classifier model in a more demanding fashion by evaluating its performance on a new batch of simulations designed to more directly examine events of the selective block. These simulations were carried out on smaller fibrotic domains (46 × 46 elements total), with single stimuli triggered separately on all four edges of the domain to increase the chance of observing unidirectional block where it might arise. The best-performing classifier model was then used to try to identify which microstructures in these new realisations of fibrosis would or would not show this type of block.

**Figure 9:**
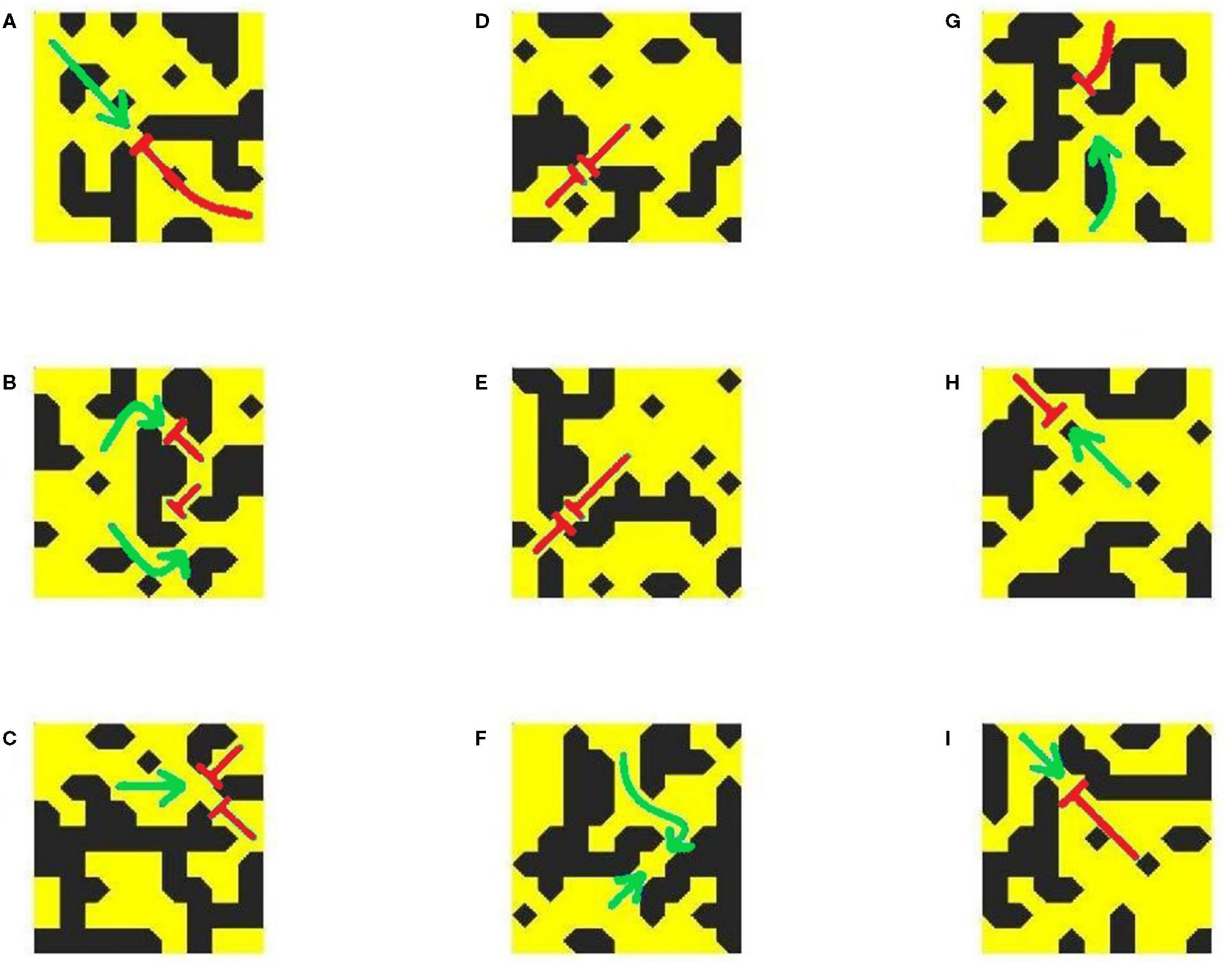
Conduction patterns in completely unseen structures from new simulations, and the corresponding predictions of the classifier model. Shown are examples of correctly identified pro-arrhythmic **(A–C)** and non-arrhythmogenic **(D–F)** micropatterns, and undetected pro-arrhythmic **(G–I)** micropatterns. All are of size 9 × 9 elements. Green arrows indicate the directions of AP propagation, with red flat arrowheads indicating conduction block. Notably, the classifier model can successfully identify structures that result in a complete block from all directions **(D,E)** but could not successfully identify all pro-arrhythmic structures, particularly those where block occurs near the micropattern boundary **(H,I)**.

Figure 9 shows a range of example patterns, including those (both susceptible and not susceptible to unidirectional block) that the classifier model successfully identified, and some of the pro-arrhythmic structures that the model failed to detect. The same archetypal structure of channels connecting to open regions to produce unidirectional block is observed, although again identification by eye is significantly challenging. For example, structures exhibiting omnidirectional block (Figures 9D,E) do not seem to be immediately separable from those exhibiting unidirectional block (Figures 9A–C,G–I), but only the latter structures are able to initiate a re-entry. Our classifier model allows for the identification of this property beyond a simple human search for the obvious, qualitative patterns.

However, some patterns that show unidirectional block when simulated were not detected by the NN classifier, despite its high accuracy on the data originally used to test its performance. There could be several reasons for this. The unidirectional block events observed in false-negative cases often occur very close to the micropattern boundary (Figures 9H,I). In such cases, there is insufficient information about the structure around the wavefront at the critical location of the block, and so the classifier model struggles to predict it. Additionally, in these smaller-scale simulations, many more of the micropatterns evaluated for testing will fall closer to the domain boundaries, where the balance of source and sink can be affected by the initial stimulus and the inability of travelling wavefronts to form their full ‘tail' of activated cells that provide an additional electrotonic sources of depolarisation. This is likely due to the fact that the structure responsible for conduction block (unidirectional or otherwise) will not precisely coincide with the location where the wavefront dies out. We discuss this further in Conclusions.

## 4. Conclusions

We have used high-throughput simulation to approach an exhaustive exploration of the issue of re-entry initiation in fibrosis-afflicted tissue, a key precursor to arrhythmia (Hansen et al., 2015; Sachetto Oliveira et al., 2018a). It is known, at least for randomly placed obstructions as considered here, that the probability a site is obstructed is a critical determinant of re-entry formation (Vigmond et al., 2016; Sachetto Oliveira et al., 2018b). This finding was recapitulated in this study, for a different type of computational mesh and was extended by also exploring how different patterns of activation interact with these regions of afflicted tissue. In particular, we have demonstrated that for the most risk-associated extents of fibrosis (ρ~0.49), a majority of fibrotic realisations were in fact capable of initiating re-entry from a single stimulus but only for waves sourcing from a select few pattern-specific locations. This suggests that lower rates of initiation previously reported (Sachetto Oliveira et al., 2018b) are largely a function of only a single stimulus pattern being considered in that study. This additionally sheds light on one role of ectopic beats in arrhythmia initiation; if one of the stimulus scenarios is said to correspond to a healthy sinus rhythm activation pattern, then the other stimulus scenarios are related to events such as premature contractions and can often initiate re-entry even when the typical activation sequence does not.

Although we observed activation sequence to be similarly as important as structure in terms of producing re-entrant waves that escape the scar region, the fine-scale events of selective block required to initiate any re-entrant activity were not expected to be overly dependent on activation sequence. This intuition was seen to hold, with a NN classifier model trained only using binary arrays of fibrosis occupancy (no activation pattern information) obtaining very good accuracy (up to 91% for this very challenging learning problem). We also used classifier accuracy to suggest the important length scale for identifying the unidirectional block in these fibrotic micropatterns, observing 9 × 9 patterns to best balance information content and learning problem dimensionality for the NNs. This suggests the effective length scale for individual events of unidirectional (or other selective) conduction block to be ~ 0.5 mm or a little larger.

When the classifier was tested on completely new data (new simulations not used for training, validation, or testing), it remained able to detect the key structures involved in generating unidirectional block events. Impressively, completely-blocking structures (i.e., blocking from all directions) could be correctly classified. This more challenging test of the classifier model did expose some of the limitations of the approach used in this study, however. First, our RVI-based detection method picks out the locations where activation dies out, but this does not always perfectly correspond to the structure most responsible for the failure to propagate. For example, a wavefront emerging from a thin channel into a bay of excitable tissue may die out a little way into the bay, even though the structure surrounding where the channel ends is the most important. One potential direction forward is improving the block detection algorithm, so it better localises the structure responsible for the unidirectional block instead of wave die-out points. Another direction is to move away from detecting specific sites of unidirectional block altogether, and instead attempt to classify micropatterns using data generated by simulating AP propagation across the micro patterns themselves.

As the focus of this study was purely on how much fibrotic structure itself can inform the risk of re-entry, we have not considered the importance of specific electrophysiological conditions for the initiation and sustainment of re-entrant activation patterns. Some examination of the effects of parameter variability in this context has already been carried out (Lawson et al., 2020), but it is a limitation of this study that we have not explicitly considered how different electrophysiological conditions impact the importance of structure vs. activation sequence or the ability to predict structures that selectively block. We suspect that if the conductivity of unobstructed tissue was adjusted, or a different cell model (or parameter values for the BOCF model) was used, the general conclusions we have drawn here would remain valid, but of course classifier models would need to be retrained. Anisotropic conduction, in particular, might also have a pronounced effect on our observations here, especially considering that different ‘textures' of fibrosis meaningfully act to change the effective anisotropy of afflicted tissue (Nezlobinsky et al., 2020).

We have used a generously sized region of afflicted tissue for data generation in this study, larger than the minimal size required to support re-entry in similar simulations (Sachetto Oliveira et al., 2018b) and larger than micro-re-entrant paths observed in explanted hearts (Hansen et al., 2015). Domain size certainly effects the probability of observing a sustained re-entry, but the observation that the direction of the initial wavefront is critical for re-entry initiation should be robust to the domain size. We have demonstrated that the individual micro-structures that do or do not exhibit selective or unidirecitonal block act on a length scale of about ~0.5 mm, much smaller than the size of the full simulation domain. A bigger limitation of our choice of domain is its two-dimensional nature, a necessity for carrying out the number of simulations performed here. In three-dimensions, critical length scales and fibrotic extents of highest risk would be expected to change, owing to the differences in source/sink balance (Xie et al., 2010; Sachetto Oliveira et al., 2018b).

In summary, a new pipeline was implemented to generate two datasets for pro-arrhythmic and non-arrhythmic fibrotic patterns. The pipeline involves simulations of re-entries within fibrotic substrates augmented by stimulations coming from multiple sites and the automatic identification of unidirectional blocks *via* the RVI method. These datasets were used to train and test a neural network that was able to successfully classify (accuracy up to 91%) micropatterns by only taking as input their structures. Therefore, our results suggest that machine learning provides tools that can be further exploited to address fundamental questions such as the relationship between anatomical heterogeneity and re-entry risk, and over what spatial scale this heterogeneity should be considered.

## Data Availability Statement

The raw data supporting the conclusions of this article will be made available by the authors, without undue reservation.

## Author Contributions

RH and BL created the simulation tools used. RH performed the training and testing of neural network classifiers and created tools used in visualising results. All authors contributed to the analysis of results and subsequent development of the study, original study concept, and drafting of the manuscript.

## Conflict of Interest

The authors declare that the research was conducted in the absence of any commercial or financial relationships that could be construed as a potential conflict of interest.

## Publisher's Note

All claims expressed in this article are solely those of the authors and do not necessarily represent those of their affiliated organizations, or those of the publisher, the editors and the reviewers. Any product that may be evaluated in this article, or claim that may be made by its manufacturer, is not guaranteed or endorsed by the publisher.
